# A Miniature Biomedical Sensor for Rapid Detection of *Schistosoma japonicum* Antibodies

**DOI:** 10.3390/bios13080831

**Published:** 2023-08-20

**Authors:** Shengjie Hu, Xuecheng Jiang, Liang Yang, Xue Tang, Guofeng Yang, Yuanyuan Hu, Jie Wang, Naiyan Lu

**Affiliations:** 1 School of Food Science and Technology, Jiangnan University, Wuxi 214122, China; 6210112145@stu.jiangnan.edu.cn (S.H.); 6201205015@stu.jiangnan.edu.cn (X.J.); yangliang@jiangnan.edu.cn (L.Y.); tangxue@jiangnan.edu.cn (X.T.); gfyang@jiangnan.edu.cn (G.Y.); 2 National Health Commission Key Laboratory of Parasitic Disease Control and Prevention, Jiangsu Provincial Key Laboratory on Parasite and Vector Control Technology, Jiangsu Provincial Medical Key Laboratory, Jiangsu Institute of Parasitic Diseases, Wuxi 214064, China; 3 Changsha Semiconductor Technology and Application Innovation Research Institute, College of Semiconductors (College of Integrated Circuits), Hunan University, Changsha 410082, China; yhu@hnu.edu.cn

**Keywords:** biomedical sensor, AlGaN/GaN high electron mobility transistors (HEMT), *Schistosoma japonicum*, soluble egg antigens (SEA), rapid detection

## Abstract

Schistosomiasis, typically characterized by chronic infection in endemic regions, has the potential to affect liver tissue and pose a serious threat to human health. Detecting and screening for this disease early on is crucial for its prevention and control. However, existing methods encounter challenges such as low sensitivity, time-consuming processes, and complex sample handling. To address these challenges, we report a soluble egg antigen (SEA)-based functionalized gridless and meander-type AlGaN/GaN high electron mobility transistors (HEMT) sensor for the highly sensitive detection of antibodies to *Schistosoma japonicum*. Immobilization of the self-assembled membrane on the gate surface was verified using a semiconductor parameter analyzer, scanning electron microscope (SEM), and atomic force microscopy (AFM). The developed biosensor demonstrates remarkable performance in detecting anti-SEA, exhibiting a linear concentration range of 10 ng/mL to 100 μg/mL and a sensitivity of 0.058 mA/log (ng/mL). It also exhibits similar excellent performance in serum systems. With advantages such as rapid detection, high sensitivity, miniaturization, and label-free operation, this biosensor can fulfill the requirements for blood defense.

## 1. Introduction

Schistosomiasis is an important tropical and subtropical parasitic disease, especially prevalent in poor areas lacking adequate sanitation facilities, and it is regarded as a disease that is difficult to control [1,2,3]. The disease is primarily transmitted through contact with water contaminated by schistosome cercariae during daily agricultural, domestic, and occupational activities. When the cercariae penetrate the host skin, transform into endoparasitic larvae, known as schistosomes, and migrate into the vascular system, the infection begins [4]. Globally, there are over 200 million reported cases of schistosomiasis each year, with nearly 800 million individuals at risk [5]. In Asia, *Schistosoma japonicum* is the primary source of transmission for the disease. The soluble eggs persist in the liver and give rise to granulomas, ultimately leading to liver fibrosis and host mortality [6,7].

Currently, the treatment of schistosomiasis depends on praziquantel (PZQ), which effectively kills adult worms, but cannot repair existing immunopathological damage. Concerns also exist regarding the emergence of drug resistance due to its widespread and intensive usage [8,9,10]. Thus, early screening and diagnosis remain the optimal approach to address schistosome infections, especially in high-risk areas and vulnerable populations.

The schistosome antigen system is complex, primarily because the infection in the definitive host involves multiple cycles [11]. Over the past four decades, numerous antigens have been investigated for their potential utilization in antibody detection systems for schistosomiasis. Research has revealed that the host’s immune response is predominantly targeted against soluble egg antigens (SEA) that are released by japonicum eggs [12,13]. Following infection, the antibodies (IgG) against SEA are rapidly produced in the host serum and persist even after successful treatment. This phenomenon facilitates the assessment of schistosome infection status in vivo and the evaluation of treatment efficacy. Hence, utilizing SEA as a probe and SEA-specific antibodies as diagnostic markers offers a dependable and efficient approach for the diagnosis and evaluation of schistosome infection [14,15,16].

A series of test methods have been evolved to detect schistosomal antibodies, such as the dot immunogold filtration assay (DIGFA), indirect hemagglutination assay (IHA), immunoenzymatic assay (EIA), and indirect fluorescent antibody assay (IFA) [17,18,19]. However, these methods have some common drawbacks, including low sensitivity, complex operation, long detection cycles, and expensive equipment, which make it difficult to perform early diagnosis as well as adapt to the needs of blood prevention. A rapid and convenient method is urgently needed.

In recent years, the AlGaN/GaN high electron mobility transistor (HEMT), a semiconductor device, has gained attention from researchers due to its excellent sensing performance, biocompatibility, and adhesion properties [20,21,22]. The presence of a high concentration of two-dimensional electron gas (2DEG) near its heterojunction is attributed to piezoelectric and spontaneous polarization effects. When the target binds to the gate surface, the 2DEG concentration changes, which leads to current changes [23,24]. Only a simple modification of the probe is required to produce a sensitive analytical signal without additional radioactive, fluorescent, or enzymatic labeling. Due to its high level of integration, standardization, and mass production capabilities, HEMT offers significant cost reductions in its utilization. Furthermore, the sensors eliminate the need for complex equipment and operations. These inherent advantages make them an ideal choice for schistosome detection.

In this study, an AlGaN/GaN HEMT sensor that selectively detects anti-SEA in blood was developed by immobilizing SEA in the sensing region. The gold-free bent gate AlGaN/GaN HEMT sensor showed an excellent response to the SEA antibody with a sensitivity of 0.058 mA/log (ng/mL) and a detection limit of 1 ng/mL. Currently, the device has demonstrated strong selectivity and stability within complex serum systems. Moving forward, enhancements will be made to the sensing apparatus. These advancements aim to facilitate field testing while maintaining the device’s performance.

## 2. Experimental Procedure

### 2.1. Reagents

3-aminopropyl trimethoxysilane (APTES) was obtained from Inokai Technology Co., Ltd. (Beijing, China). Hydrogen peroxide, concentrated sulfuric acid, and glutaraldehyde (GA) were purchased from Sinopharm Chemical Reagent Co., Ltd. (Shanghai, China). Bovine serum albumin (BSA) and phosphate buffer solution (PBS) were purchased from Aladdin Chemistry Co., Ltd. (Shanghai, China). The SEA and SEA antibodies were provided by the Jiangsu Institute of Parasitic Diseases (Wuxi, China).

### 2.2. Fabrication of the AlGaN/GaN HEMT Sensor

Growth of epitaxial layer structures on sapphire (Al_2_O_3_) substrates by metal chemical vapor deposition (MOCVD). Electron-beam evaporation (EBM) was used to evaporate Cr/Al/Ti/Pt/Au (1.5/120/75/50/1500 nm), which were then used as ohmic contacts and patterned via photolithography. The source and drain contacts were finally formed by annealing at 270 °C for 15 min in nitrogen. The distance between the source and drain was kept at 650 μm. To provide a protective passivation effect, a 250 nm thick SiO_2_ layer was deposited using plasma-enhanced chemical vapor deposition (PECVD). Subsequently, etching was performed to expose the source-drain and gate regions.

### 2.3. Immobilization Procedure

The gate was ultrasonically cleaned using anhydrous ethanol and deionized water (DI) in sequence to remove organic and inorganic impurities. Then, the devices were immersed in a cooled piranha solution (3H_2_SO_4_:1H_2_O_2_) for a duration of 15 min, followed by a thorough rinse using DI and drying in a nitrogen atmosphere. Next, the devices were submerged in an ethanol solution of APTES (5% vt) and allowed to react at 50 °C for a period of 24 h. After washing with DI water, 2.5% glutaraldehyde was added to react for 2 h. A volume of 2 μL containing 3 mg/mL of SEA was carefully applied onto the gate of the modified device, followed by incubation at 4 °C for 24 h to facilitate the required binding. Subsequently, the modified device was subjected to three washes using 10 mM PBS. To prevent non-specific binding, the aldehyde groups that were not occupied by SEA were passivated for 1 h with a 1% mass fraction of the BSA solution. Finally, the prepared devices were used immediately or vacuum-packed for short-term storage at 4 °C in a refrigerator.

### 2.4. I-V Characteristic Measurements

Different concentrations of SEA antibodies (1 ng/mL–2 mg/mL) were prepared in 10 mM PBS (pH 7.40). The active channel of the SEA antibody sensor was coated with 0.5 μL of the substance solution to be measured, followed by incubation at 25 °C in a light-free environment for 1 h to allow binding. Ultrapure water was then used to wash the device to remove unbound analytes. After air drying, a Keithley-4200 semiconductor parameter analyzer was used to measure the drain current (*I_d_*) and the drain-to-source voltage (*V_d_*) of the device.

### 2.5. Rabbit Serum Testing

To verify the actual effectiveness of the sensor, serum samples from different normal rabbits were used to prepare a range of concentrations of anti-SEA for testing. The normal rabbit serum samples used for testing were provided by the Jiangsu Institute of Parasitic Diseases (Wuxi, China).

## 3. Results and Discussion

### 3.1. Fabrication and Characterisation of AlGaN/GaN HEMT Sensors

**Figure 1:**
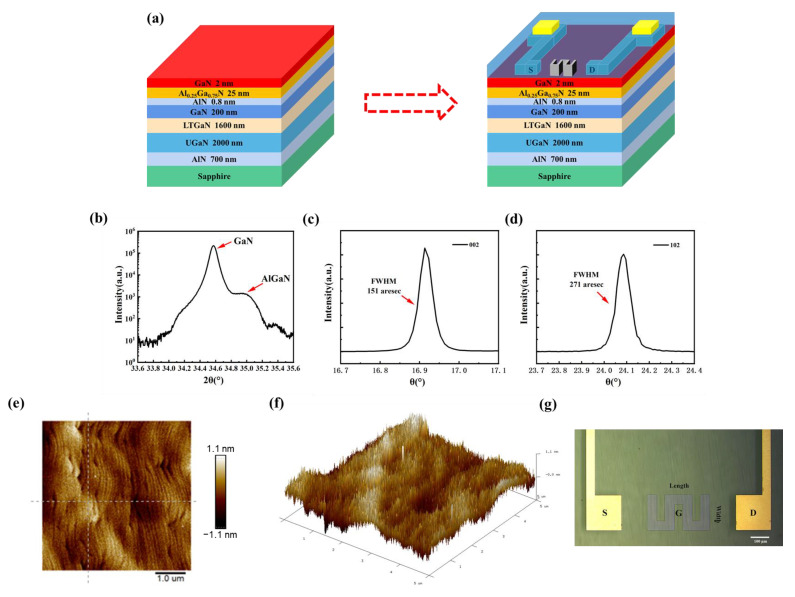
(**a**) Main fabrication parts of the AlGaN/GaN HEMT. High-resolution X-ray diffraction scans of the (**b**) AlGaN/GaN plane, (**c**) GaN (002), and (**d**) GaN (102). (**e**) 2D and (**f**) 3D AFM image of the epitaxial layer. (**g**) Top-view photomicrograph of the sensor.

The HEMT device preparation is divided into two main parts: epitaxial layer stacking and source-drain electrode preparation (Figure 1a). In this study, an affordable and durable sapphire substrate is employed. To reduce the interfacial tension caused by lattice mismatch, a thin transition layer of AIN with nanometer-scale thickness is introduced. At the same time, a 2000 nm thick undoped GaN and 1600 nm thick low-temperature GaN buffer layers are necessary to minimize the film defects induced by strain accumulation during epitaxial wafer growth. The GaN channel layer with a thickness of 200 nm is used to guarantee that the domain-limiting property of the two-dimensional electron gas (2DEG) is not reduced and the carrier mobility is improved. In order to establish an energy barrier that aids the passage of carriers, an AlGaN material with an aluminum fraction of 0.25 is utilized. The appropriate thickness of AlGaN material is beneficial to improve the saturated output power as well as the signal gain characteristics. Additionally, a 0.8 nm thick AlN separator layer is used to reduce the effect of ion scattering on the 2DEG mobility and concentration in the channel. A 2 nm GaN cap layer is introduced, which helps in reducing gate leakage current by leveraging polarization effects to enhance the effective barrier. Finally, silicon dioxide is used as the passivation treatment material to further enhance the channel electron concentration of the HEMT [25,26,27].

A key factor governing the performance of AlGaN/GaN HEMT devices is the quality of the epitaxial film. Therefore, the non-destructive evaluation of material suitability at the material growth level using X-ray diffraction (XRD) is required to ensure reliable device performance. By scanning over a wide range of angles (ω-2θ), the diffraction angles corresponding to the individual components can be obtained. The XRD scanning curves of the GaN (002) film are shown in Figure 1b. Two major diffraction peaks (34.57° and 34.98°) corresponding to GaN and Al_0_._25_Ga_0_._75_N can be observed. Subsequently, a scan is conducted at a predetermined diffraction angle, allowing for the collection of diffraction peak information pertaining to specific crystal surfaces. The XRD images of GaN 002 and 102 are depicted in Figure 1c,d, respectively. The extracted full-width half maximum (FWHM) of these diffraction peaks provided insights into dislocation density and stresses. By fitting a Gaussian function, the FWHM of GaN (002) is determined to be 151 arcsec, whereas the FWHM of GaN (102) is calculated to be 271 arcsec. The smaller values of the FWHM indicate a higher degree of crystal quality in the epitaxial layer film.

Furthermore, the thickness, roughness, and surface morphology of the epitaxial structure were examined using atomic force microscopy (AFM). The surface roughness (Ra) was 0.248 nm and the root mean square (Rq) was 0.316 nm in the scanning range of 5 μm × 5 μm (Figure 1e). To visualize the microstructure more, a three-dimensional (3D) scanning map is shown in Figure 1f. The typical stepped phase profile implies good uniformity and small strain during the growth of the epitaxial structure.

After passivation and gate preparation, the sensing element is finally examined via the optical microscope (Figure 1g). The gate length is 200 μm and the width is 50 μm. Through three meanderings, the sensing area expands to 35,000 μm^2^. In comparison to the traditional gate, the meandered gate exhibits a more uniform distribution of the built-in electric field. By employing a gridless structure, which means no additional metal vaporization is required for the gate area, the modifier can be directly bonded to the gate. The approach reduces the distance between the surface charge and 2DEG, resulting in an enhanced detection sensitivity.

### 3.2. Functionalization and Characterization of AlGaN/GaN HEMT Sensors

**Figure 2:**
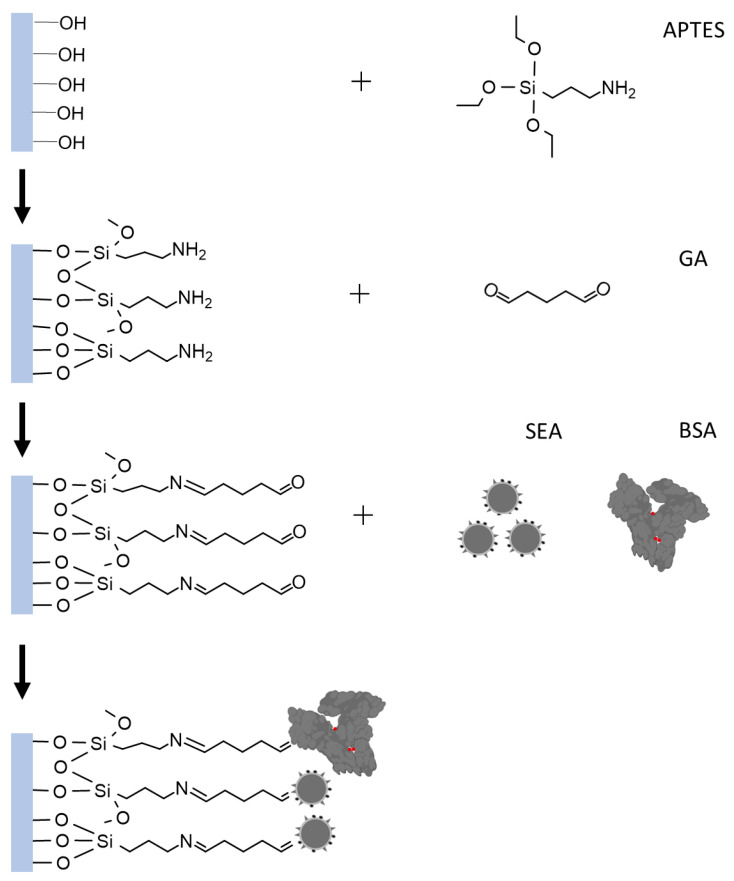
Illustration of SEA functionalization in the gate region.

The immobilization of SEA is mainly based on APTES silylation and GA activation. To ensure the formation of an effective APTES layer, the surface where the biomolecule will be immobilized must be hydrophilic, containing reactive hydroxyl groups. Therefore, a piranha solution processor device was used to achieve the purpose of removing the oxide layer on the sensing region surface and introducing enriched –OH [28,29]. Following APTES silanization, the sensing region was further enriched with –NH_2_. The linking of GA to the matrix occurred via –NH_2_ terminal groups. The other free terminal of –CHO groups is attached to the amino group of SEA. This reaction is primarily based on the Schiff base reaction or Michael addition [30]. The BSA molecule’s surface amino groups react with activated carboxyl groups, blocking excessive active sites. Simultaneously, its hydrophilic carboxyl groups can help deter nonspecific protein adsorption, according to Berg’s law. Therefore, BSA enhances the sensor’s antifouling performance [31]. The specific process of modification is shown in Figure 2.

**Figure 3:**
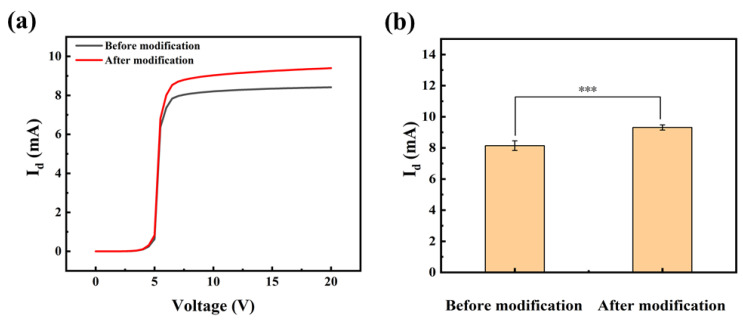
(**a**) The I-V curves prior to and following modification, along with (**b**) the significance analysis. Error bars indicate the standard deviation; *** indicates *p* ≤ 0.001 (Welch’s *t*-tests).

Whether the modification process is successful or not directly affects the ability of HEMT devices to recognize and bound anti-SEA. The effectiveness of the modification strategy was validated by I-V characterization. The presence of a molecular layer bound to the device surface was established by comparing the I-V characteristics of the gate before and after the modification. Figure 3a exhibits typical examples of sigmoidal-shaped I-V curves for devices measured before and after modification. Specifically, within the drain voltage range of 0–5 V, known as the initial zone, there is a minimal change in *I_d_*. In the range of +5–7 V, referred to as the ascending zone, the *I_d_* undergoes rapid changes. Subsequently, within the range of +7–20 V, known as the saturation zone, the current gradually increases until it reaches saturation. Importantly, the modification process did not result in any damage to the device, and its electrical characteristics remain within normal parameters. The phenomenon of a significant increase in current after modification was also common in other samples. The results of numerous experiments were meticulously analyzed for significance, and a substantial difference before and after the modification was determined with a calculated *p*-value of 0.0005 (Figure 3b).

**Figure 4:**
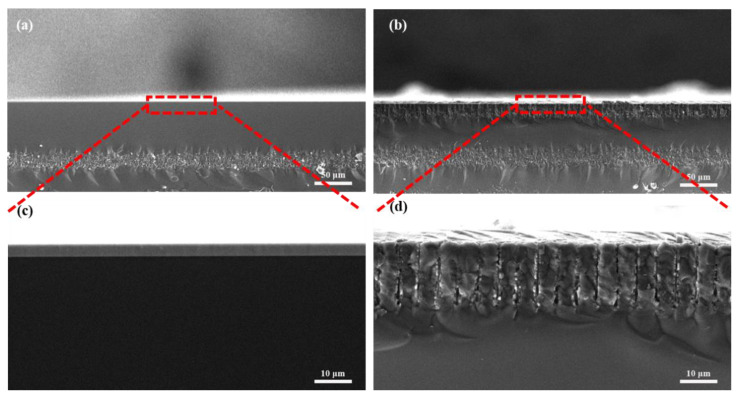
Cross-sections and enlargements of unmodified devices (**a**,**c**) and modified devices (**b**,**d**).

The attachment of SEA was confirmed using the scanning electron microscope (SEM). Figure 4a displays a cross-sectional image of the unmodified sensor, revealing a remarkably smooth surface even when magnified (Figure 4c). High-resolution SEM images in Figure 4b,d illustrate the cross-section of the SEA-modified device. The images demonstrate the presence of an undulating surface on the sample, along with discernible laminated modifications. These observations serve as compelling evidence of the successful modification of the probe onto the gate surface.

**Figure 5:**
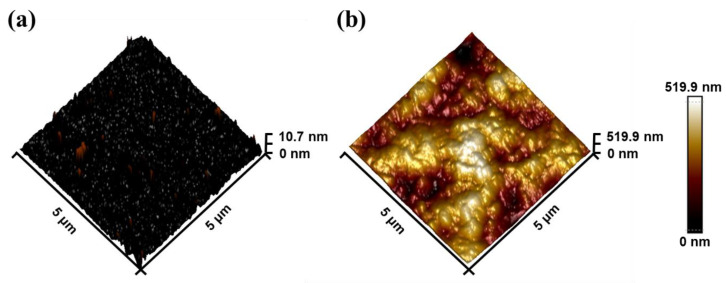
The AFM height images of modification (**a**) before and (**b**) after.

To further reflect the surface modification, AFM technique characterization was also performed. Prior to modification, the surface exhibited a relatively flat profile, Ra = 0.599 nm and Rq = 1.18 nm (Figure 5a). However, after modification, the surface morphology, as depicted in Figure 5b, displayed noticeable bumps resulting from SEA stacking. The modification led to an increase in some parameters, such as roughness and root mean square (Ra = 61.4 nm, Rq = 76.6 nm).

### 3.3. Detection of SEA Antibody in PBS Buffer

**Figure 6:**
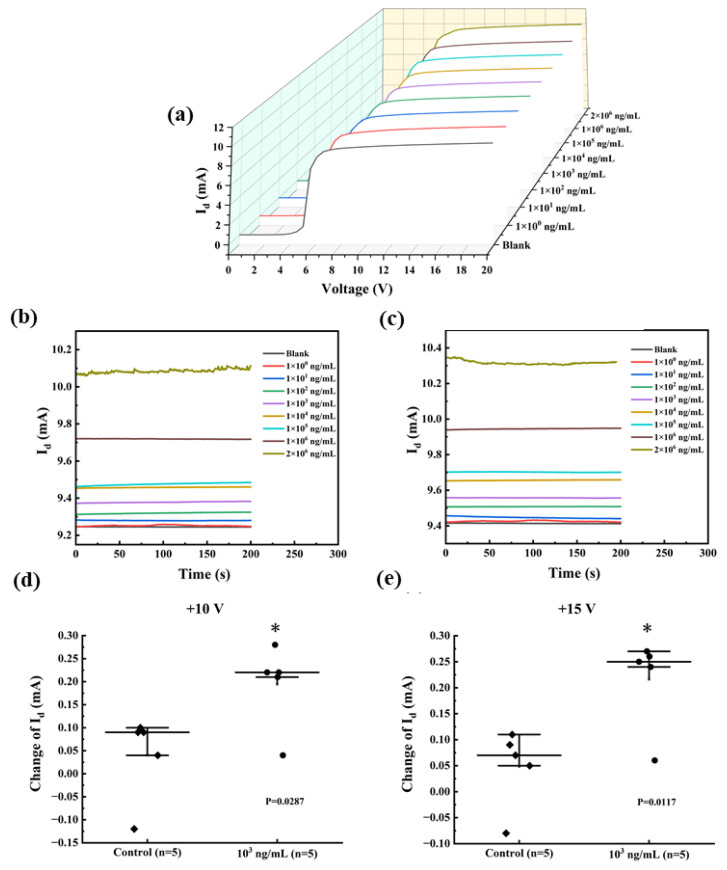
(**a**) The I-V curves for various concentrations of anti-SEA in the range of 0–20 V. The value of *I_d_* changes when using SEA. I-T response of the sensor at (**b**) +10 V and (**c**) +15 V. Functionalized AlGaN/GaN HEMT sensors were incubated with 10^3^ ng/mL SEA in PBS (●) and non-spiked PBS (◆) at the measurement voltage (**d**) +10 V and (**e**) +15 V. Error bars indicate the standard deviation; * indicates *p* ≤ 0.05 (Welch’s *t*-tests).

After validating the device modifications, the response behaviors of the anti-SEA biosensor in PBS were measured. Measurements on the I-V behavior of the biosensors were conducted using different anti-SEA concentrations, as shown in Figure 6a. When the leakage voltage (*V_d_*) was a fixed value in the +7–20 V interval, the *I_d_* showed a gradual increase with increasing concentration of the substance to be measured. Therefore, a fixed drain voltage was chosen as the detection condition within the saturation region. The breakdown voltage (BV) of the AlGaN/GaN HEMT is lower than its theoretical limit due to the premature breakdown at the gate edge caused by E-field crowding [32]. In addition, there is fault tolerance in the actual fabrication, modification, and application process, and it is not appropriate to choose the voltage at the two ends of the interval as the test condition. Taken together, +10 V and +15 V in the middle of the saturation interval were used for subsequent testing.

The current-time (I-T) dependence curves of HEMT devices reflect important properties such as sensor response time (with or without delay), stability (whether it fluctuates or not), and dynamic range (test limit). Constant testing at +10 V and +15 V for 200 s, the results are shown in Figure 6b,c, respectively. When the *V_d_* was set to +10 V, the minimum observed *I_d_* was 9.24 mA, whereas the maximum *I_d_* was 10.10 mA. These values corresponded to anti-SEA concentrations of 0 and 2 mg/mL, respectively. As the anti-SEA concentration varied from 0 mg/mL with a bias voltage, the current increased from 9.41 mA to 10.32 mA. At both biases, the I-T curves corresponding to 1 ng/mL of the to-be-tested material were difficult to distinguish from the PBS solution, implying a possible lower limit of detection. After adding 2 mg/mL of the solution to be tested, the I-T curves exhibited minor fluctuations, which remained within acceptable limits. In conclusion, the sensor demonstrates superior performance with its fast response time (approximately 50 s) and wide testing range (ng/mL-mg/mL), suggesting the potential for qualitative detection. Significance was also compared between spiked (10^3^ ng/mL) and unspiked samples (control) (Figure 6d,e). The results showed that the significant difference at +15 V (*p* = 0.0117) was greater than +10 V (*p* = 0.0287), indicating a greater change in *I_d_*. Therefore, the standard curve was tested and fitted at +15 V.

**Figure 7:**
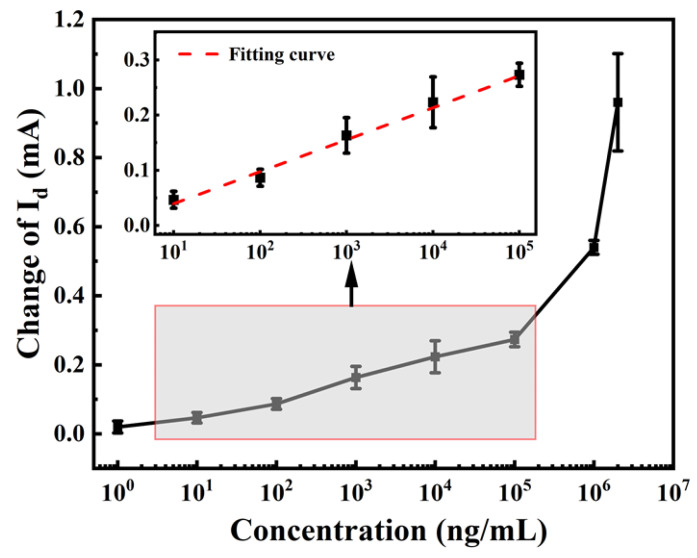
Measured and fitted curves describing the relationship between current response and anti-SEA concentration.

The mean values of the output signals during the test time were calculated as the final test values. To ensure accuracy, each concentration was repeated three times. The standard curve of the schistosome sensor in a PBS solution was established by plotting the amount of current variation against the logarithm concentration of the substance to be tested, as illustrated in Figure 7. The *I_d_* of the sensor exhibited a linear correlation with the concentration of anti-SEA within the range of 10–10^5^ ng/mL. Fitting the linear interval led to the following calibration equation: ΔI_d_ = 0.058lgX − 0.018, with a correlation coefficient of 0.991. According to the definition provided by the International Union of Pure and Applied Chemistry (IUPAC), the analytical sensitivity can be expressed by the slope of the sensor input–output calibration curve [33]. Therefore, the resulting sensor sensitivity was 0.058 mA/log (ng/mL).

The operating mechanism of the sensor can be explained using the capacitance model. The fluctuation in the drain current signal is associated with the capacitance of the solution being measured. To enhance comprehension, this relationship can be expressed using the following equation:

(1) $$V_{g}=\Delta V_{s}+\Delta V_{cx}$$

(2) $$\Delta V_{cx}=\frac{C_{S}}{C_{S}+C_{cx}}\times V_{g}$$

where *V_g_*, ∆*V_s_*, and ∆*V_cx_* represent the gate voltage, the voltage drop of the test solution, and the voltage drop of the dielectric, respectively. *C_s_* denotes the solution capacitance, and *C_cx_* is the dielectric capacitance. According to Equation (1), the variation in gate voltage is influenced by the voltage fluctuations of both the solution and the dielectric (AlGaN). The potential drop of AlGaN is related to the solution capacitance *Cs* (Equation (2)). A higher *Cs* results in a higher effective *V_g_* applied to the dielectric of the device, leading to a greater increase in *I_d_*. The *C_s_* changes when surface functionalization or probe-target binding occurs. Therefore, it is possible to detect and monitor biological interactions on the sensor surface using this device [34,35].

### 3.4. Analysis of Serum Systems

**Figure 8:**
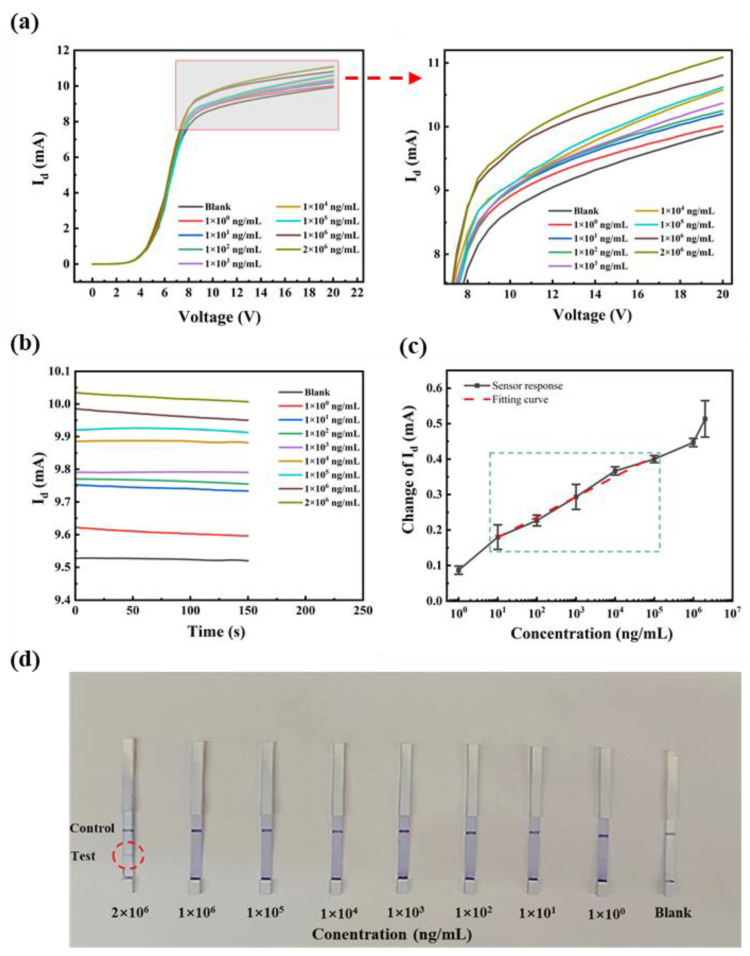
Rabbit serum: (**a**) the I-V curves of different concentrations of anti-SEA at 0–20 V; (**b**) the I-T response at +15 V; (**c**) fitting curves; and (**d**) commercially available kit assay results.

The SEA antibodies are mainly found in the serum. In order to examine the detection performance of the anti-SEA biosensor in real samples, different concentrations of anti-SEA were added to normal rabbit serum to form complex samples. The test results were verified using I-V and I-T curves complementing each other. As shown in Figure 8a,b, the *I_d_* increased with the gradual increase in doping concentration. The biosensor exhibited a consistent pattern when detecting anti-SEA in both the PBS and rabbit serum, implying that the observed current change is attributed to anti-SEA. A more careful comparison revealed that the sensing behavior in serum was more complex than in PBS, which showed large changes in amplitude and errors. This may be due to the more complex composition of the serum, which contains hundreds of different proteins and metabolites [36]. Based on the analysis of multiple experiments, a relationship curve depicting the correlation between the magnitude of the current change and the logarithmic concentration of the target substance was plotted, as illustrated in Figure 8c. When the concentration of the test substance was 10–10^5^ ng/mL, the standard curve was linear with the fitting equation ΔI_d_ = 0.057 lgX + 0.12 and the linear regression coefficient was 0.975. The linear range and sensitivity were basically the same as that of the PBS system. The Schistosoma antibody detection kits available in the market were also subjected to testing and comparative analysis (Figure 8d). The concentration increased sequentially from right to left, and the test strip was positive (two bars) when the concentration was 2 mg/mL. In comparison, our sensor has lower sensitivity.

**Figure 9:**
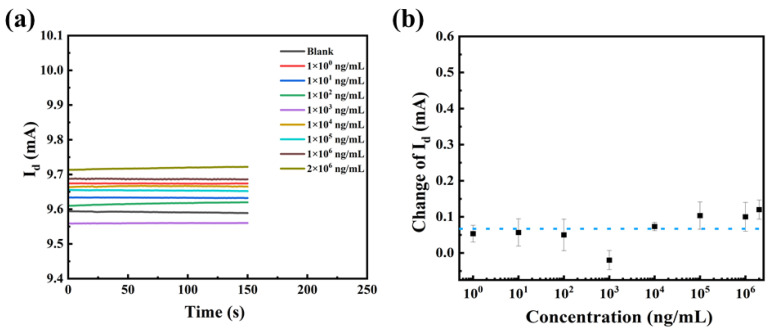
(**a**) I-T curves and (**b**) the current changes in the presence of different concentrations of anti-SEA.

To verify the reason for the change in the device current, devices with unmodified SEA were prepared for testing (other steps unchanged). The I-T signals of the unfunctionalized devices were very smooth for all concentrations, which was attributed to the fact that no antigen-antibody binding reaction occurred on the surface of the devices (Figure 9a). Testing results were quantitatively analyzed, and are shown in Figure 9b. The response of the device to the substrate to be tested fluctuated around 0.064 mA with no regularity. This phenomenon is caused by the background noise of the serum matrix. By comparing the responses of the unfunctionalized and functionalized device, it can be demonstrated that the biosensor’s reaction to the test article is reliant on specific antigen-antibody recognition.

In addition, a comparison with existing assays was made to better explain the advantages of the present work, and the results are shown in Table 1. The proposed sensor is superior to others in terms of detection limits and response speed. It is worth mentioning that the HEMT sensor does not require additional reference electrodes or complex systems, allowing for on-site testing in difficult environments.

## 4. Conclusions

In the present study, a label-free biosensor based on SEA-functionalized AlGaN/GaN HEMT was designed, characterized, and tested. When exposed to the target solution, the current signals of the biosensors showed highly robust and reproducible variations. The detection limit of 1 ng/mL in both PBS and serum systems is better than that of the commercial kits. In addition, the unmodified SEA sensor has minimal irregularity in response to the different concentrations of SEA, confirming its excellent selectivity. Other important advantages of the biosensor are the wide linear detection range of 10 ng/mL–100 μg/mL and the assay time of less than 200 s. It is suitable for rapid detection and scale-up screening of at-risk populations, as well as for patient healing monitoring. Lastly, the availability of species-specific antigens enables the implementation of this biosensing concept, simplifying and enhancing the fast detection and identification of various parasites. This approach effectively overcomes disadvantages, such as inefficiency and inconvenience.

## Figures and Tables

**Table 1:** Comparison of the analytical performances of the proposed biosensor with other biosensors for the detection of Schistosoma sp.

Assay Format	Assay Time	Labelling or Signal Amplification	Immunosensor Type	Linear Range	Lowest Concentration of Anti-SEA	Reference
Sandwich immunoassay	~20 min	4-chloro-1-naphthol and HRP	mass piezoelectric immunosensor	10–100 ng/mL	5 ng/mL in PBS	[37]
Sandwich immunoassay	~20 min	Cu, AuNP	magnetic electrochemical immunosensor	2 ng/mL–15 μg/mL	1.3 ng/mL in spiked serum	[38]
Sandwich immunoassay	/	HRP and AuNP	metalloimmunosensor	6.4 ng/mL–100 μg/mL	3 ng/mL in PBS	[39]
Sandwich immunoassay	<15 min	rSPG-RFP	fluorescence immunochromatographic strip	/	1:10000 in serum	[40]
Direct Immunoassay	<200 s	Label-free	AlGaN/GaN HEMT	10–10^5^ ng/mL	1 ng/mL in PBS buffer and spiked serum	This work

## Data Availability

The datasets generated and analyzed during the current study are available from the corresponding author upon reasonable request.
